# Book Reviews

**Published:** 1801-02

**Authors:** 


### CRITICAL RETROSPECT
### OF
### MEDICAL AND PHYSICAL LITERATURE.

[ FOREIGN AND DOMESTIC. ]

---

*Geschichte der Chemie*; i. e. History of Chemistry, from the Restoration of Sciences to the end of the Eighteenth Century, by J. F. Gmelin, Professor at Gottingen, Vol. I. 1797, pp. 777, 8vo. Vol. II. pp. 790. Vol. III. pp. 1287.—1798, 1799. Gottingen, for Rosenbush.

We ought to apologize for being so late in acquainting our readers with a work that may undoubtedly be considered as a very acceptable present to chemists. The author is a man sufficiently known by the extent of his literary knowledge in chemistry, and by the superiority of his learning, which, beside his particular merits as a chemist, render him chiefly calculated for the composition of such a valuable work; and in considering the labour and indefatigable diligence that is bestowed upon it, and which few men, even when furnished with an equal stock of abilities, will hardly undergo, we should sincerely acknowledge the merits which Mr. Gmelin has acquired by that undertaking, and willingly confer on him that praise which he so justly deserves. We cannot, therefore, but disapprove the criticism that is written on it by a German Reviewer, who seems not to conceive how indulgently such a laborious work ought to be contemplated, and how slight defects, and trivial in-accuracies, are absorbed and lost in general excellence.

*In the preface*, the author acquaints his readers, that it is his intention, as a true historian, to be partial to no sect, but to follow the paths of truth and impartiality, and neither blindly to adhere to the old doctrines, nor implicitly admire the innovations with contempt of the old,

The

*The introduction* presents, in agreeable language, a sketch of the progress and changes which chemistry has experienced from its infancy to the present age, and of its state in the different periods and junctures of time. The history of chemistry may be divided *into two chief periods*, *that* which precedes the institution of learned Societies, comprehending the twelfth and the following centuries, and *that* from the seventeenth century to the present times. In the first time of the former period, chemistry was merely in the hands of the Arabs, which the author, therefore, calls *the age of the Arabs.* The Europeans, however, who followed them, being instructed in their schools, retained, for a long time, their method, doctrines, and prejudices; on which account he stiles it *the age of the Arabists.* This was succeeded by the *scholastic age*, when every science was treated and cultivated after the scholastic manner. The fourth age is that of *Paracelsus*, when that adventurer obtained a superior influence in medicine and chemistry. A great animosity prevailed about that time between the followers of Paracelsus and those of the old doctrines, which parties some enlightened men endeavoured to reconcile; and thus arose, in the first quarter of the seventeenth century, *the age of the Eclecticks;* but as Francis Sylvius de la Boë acquired a particular fame by his doctrines, *the age of Sylvius* was constituted in the second quarter of the seventeenth century. The second chief period is likewise divided into several sections; but the following survey will show the order after which the whole is disposed.

I. MIDDLE HISTORY, from the twelfth century to the middle of the seventeenth. 1. *Age of the Arabs*; twelfth century, with a part of the thirteenth. 2. *Age of the Arabists*; the rest of the thirteenth century to the beginning of the fifteenth. 3. *Scholastic age;* fifteenth century to the beginning of the sixteenth. 4. *Age of Paracelsus*; the rest of the sixteenth century. 5. *Age of the Eclecticks*; first quarter of the seventeenth century. 6. *Age of Francis Sylvius de la Boë*; second quarter of the seventeenth century.—All this comprehends the first volume.

II. MODERN HISTORY, from the middle of the seventeenth century, to the end of the eighteenth. 1. *Age of Robert Boyle*; from the middle of the seventeenth century, to the end of it. 2. *Age of George Ernest Stahl*; from the end of the seventeenth century to the last quarter of the eighteenth; contained in the second volume. 3. *Age of Lavoisier*; from the last quarter of the eighteenth century, to the present times, contained in the third volume; but, by a mistake of the printer, a part of the former age has got into this volume.

The author has given an additional value to his work by the accurate and complete quotations of the different writings of all ages; and, in this respect, it may be considered as the completest repertory for chemical literature.

---

Triumph

*Triumph der Heilkunſt*; i. e. Triumph of Medicine, or practical Inſtruction for ſuccouring the moſt deſperate morbific Symptoms, explained by Facts: A Repertory for Phyſicians and Surgeons. Edited by Dr. Chr. Aug. Struve, Practitioner at Goentitz, Vol. I. Breſlau, Hirshberg and Liſſa, for Korn the Elder, 1800, in 8vo. (price 1 rixd. 8 gr. or about 4s. 6d.)

The author explains the title of this publication in the preface as "A collection of hiſtories of diſeaſes, which, under the greateſt dangers, and moſt unfavourable ſymptoms, ended in a moſt favourable way; inſtances of the application of heroic remedies and great operations, and of the perſeverance, preſence of mind, and courage of phyſicians in the moſt deſperate diſeaſes." The inſtances and caſes are, for this purpoſe, collected from old and modern medical writings, and the author intends to increaſe them, in caſe his plan ſhould meet with approbation. He profeſſes himſelf, that a great deal of Triumph, in moſt of thoſe caſes, is more owing to Nature than to Art. It were to be wiſhed, the author had followed a better order in executing his plan, which, however, is ſomewhat retrieved by an alphabetical regiſter, added at the end of the book.

---

*Grundriſs der Chemie*; i. e. Elements of Chemiſtry, after the neweſt Diſcoveries, and calculated for the Uſe of Lectures; by Fred. Aler. Charles Gren, Profeſſor at Halle, Vol. I. ſecond edition. Halle. 1800. 8vo. (price 1 rixd. or about 3s. 6d.)

The firſt edition of this excellent book is known to our readers by a good tranſlation into Engliſh. The preſent one, which has been reviſed, after the death of the author, by Mr. Karſten, at Berlin, has undergone but few alterations, either relating to ſome later diſcoveries, or containing ſome little corrections. Thus, for inſtance, the auſtral earth is omitted amongſt the elementary earths; in lieu of which, the glycine, according to Vauquelin's diſcovery, is adduced. The ſecond part is to follow in a ſhort time.

---

*Traité du Goitre et du Cretiniſme, precédé d'un Diſcours ſur l'Influence de l'Air humide ſur l'entendement Humain*; *i. e.* Treatiſe on the Wen (Bronchocele) and Cretiniſm, preceded by a Diſcourſe on the Influence of moiſt Air on human Underſtanding. By F. E. Fodere, &c. Paris, for Bertrand, year 8, 1800, pp. 248, in 8vo.

The author, who is already known by his inquiries into the above diſeaſes, gives, inſtead of a preface, a previous explanation of the word *Cretiniſm*: "The wen (goitre), he ſays, is ſufficiently known, becauſe it is met with in every country; but not every reader will probably underſtand what the word *Cretiniſm* ſignifies. It is derived from *Cretin*, a name that is given in certain countries to individuals who are perfectly ſtupid, or fools, and who are likewiſe called idiots, &c. The word *Cretin* itſelf comes from Chretien, bon

Chretien

Chretien (Chriftian, good Chriftian) a title given to thofe idiots, becaufe they are thought not to be able to commit any fin. I have adopted this appellation in preference to any other, becaufe the individuals to whom it belongs, are more than idiots, and deferve a particular name."

In the introductory difcourfe, the Author examines the influence of moift air on the human underftanding. The Treatife itfelf is divided into four fections. *The first Section* treats of the wen or bronchocele (goitre) of the countries where it is found, of the individuals who are moft expofed to it, of the caufes by which it is produced, and of the different remedies. The Author thinks the remote caufe to be the humidity of the atmofphere, and the proximate caufe a compreffion or obftruction of the muciferous canals of the thyroid gland. In *the second Section,* Mr. Fodéré gives the hiftory of the complete and incomplete Cretinifm, which is propagated by generation, particularly by the father. The object of *the third Section* is an enquiry into the general caufe of Cretinifm and Bronchocele. After having given the topographical and meteorological defcription of thofe valleys where thefe difeafes particularly occur, and propofed the different hygrometric experiments made for that purpofe in different places of the valley of Aofta, the Author ftates, that the moifture of the atmofphere is likewife the caufe of Cretinifm; and he endeavours to refute fome other opinions relating to this difeafe, viz. of cold, of difeafes of the fkin, of drunkennefs being the caufe of Cretinifm. In *the fourth Section* the Author fhows the phyfical and moral remedies that may be employed in preventing the Wen and Cretinifm. Although thefe difeafes feem at firft fight to be peculiar to the nature of the foil, yet by obferving that the number of Cretins has confiderably decreafed in feveral places, in confequence of fome natural revolutions, and other changes produced by the hands of men, one is entitled to think, that it is poffible to ftop the farther progrefs of Cretinifm by following the rules which experience has furnifhed, and which the Author explains in this fection; where, at the fame time, he propofes a plan of regimen capable of contributing to diminifh the number of thofe unhappy creatures. We cannot but fincerely wifh, on account of the extreme degradation of human nature in the Cretins, that the philanthropic ideas of the author may be every where realized.

The diminution of the number of *Cretins,* is particularly to be attributed to the draining of the fens, to the more healthy diftribution, and fituation of the huts and cottages, the clearing of the woods, &c. by which a confiderable change in the temperature is produced; and, finally, to the progrefs which of late has been made in education and civilization. The author indicates the proper proceedings for diminifhing the moifture of the atmofphere of the Sub-Alpine valleys, and the means of ftrengthening the body againft its noxious influence. The laft chapter treats of the moral education which is moft proper to the inhabitants of the Alpine valleys, and on the means of improving the fituation of the the unhappy Cretins, and of making the Half-Cretins uſeful to the community. We conclude the account of this work by remarking, that it is undoubtedly claſſical on thoſe diſeaſes, and particularly on that deficiency of the human underſtanding, to which the appellation of Cretiniſm is aſſigned, and which ſeems to be indigenous and endemic in the valleys of the Italian and Swiſs Alps.

---

*Hiſtoire Naturelle des Salamandres de France ; précédée d'un Tableau Méthodique des autres Reptiles indigènes :* i. e. Natural Hiſtory of the Salamanders of France, preceded by a methodical Table of other indigenous Reptiles, with ſix coloured plates. By P. A. Lacretelle, Aſſociate of the National Inſtitute, &c. Paris, for Villers, year 8, 1800, in 8vo. pp. 47 and 61.

Cit. Lacretelle had already preſented, in the year 5, 1797, to the Mathematical and Phyſical Claſſes of the National Inſtitute, a Memoir on the Salamanders of France, which was very favourably received, and ordered by the National Inſtitute to be printed with the Memoirs of the Foreign Literati. Since that time, however, the author has made new inquiries, of which he has publiſhed the reſults in the work we are here announcing. It is divided into two parts; in *the firſt* of which, Cit. Lacretelle propoſes a methodical arrangement of the other indigenous reptiles, after the claſſification of Cit. Brongniart. In *the ſecond,* he proceeds to the natural hiſtory of the ſalamanders of France. After having mentioned the modern naturaliſts who have written upon this ſubject, and ſhowed how unſettled is the nomenclature of theſe animals, what they ought to take in the ordines naturales, the author treats of their organization and œconomy, compared with that of other reptiles; of their retirement and re-appearance; and inquires how far we know the generation of the terreſtrial ſalamander; and then proceeds to ſpeak of the act of generation among the aquatic ſpecies, on the diſpoſition of their eggs, &c. on their developement, &c. and on their faculty of regenerating the limbs which they accidentally loſe, or are taken from them.

Cit. Lacretelle terminates this part with remarks on the ſexual difference of ſalamanders, on their organs of voice, and other peculiarities. He exhibits a methodical table of the ſalamanders of France, in French and Latin, and concludes the whole with a deſcription of the ſpecies. Theſe are, 1. *La ſalamandre terreſtre,* which is figured in the firſt plate; and of which the ſecond exhibits an accurate figure of the ſkeleton. 2. *La ſalamandre marbrée.* 3. *La ſalamandre cretée,* i. e. the creſted ſalamander. Theſe two ſpecies are repreſented on the third plate; and the fourth exhibits the female of the creſted ſalamander with opened belly, to ſhow the ſituation of the inteſtines. 4. *La ſalamandre abdominale,* engraved in plate 5, fig. 4, on which likewiſe two young ones, in their different ſtate, are repreſented. 5. *La ſalamandre ceinturée,* with a girdle, figured plate 5, fig. 5. 6. *La ſalamandre ponctuée,* or the dotted ſalamander, of which male and female are figured plate 6, fig. 6. A. and fig. 6. B. 7. *La ſalamandre palmipède,* engraved graved plate 6, fig. 7. and a variety of it with its feet more palmipede, fig. 7, B.

### *Prof. Reich's Theory and Practice of Fever.*

[Continued from Vol. IV. pp. 556—563.]

"Since that time," Mr. Reich proceeds, "I became more acquainted with the great ufe of mineral acids, and employed them in more or lefs quantity in all cafes of fever, whatever appellation they may have, acute, remittent, fimple, complicated, idiopathic or fympathic, fymptomatic or original, acceffory, inflammatory, putrid, pituitous, bilious, exanthematous, nervous, lantefcent, epidemic, contagious, &c. I was convinced that they alone are capable of curing every fever, in the fhorteft fpace of time which can naturally be imagined, and of removing all danger, when no organic lefion exifts in any part of the body, and no error is committed either by the phyfician, or the patient and thofe who attend him.

"Having for a long time only ufed the fulphuric acid, and often experienced that the patients did not relifh it, nor that it acted fpeedily enough, on account of its not poffeffing that degree of volatility to emit the oxygen quick enough, and of its caufing fome inconveniencies to the ftomach, I made the experiment of giving the muriatic acid internally, in the fame quantity, and even in larger dofes, being more volatile than other acids; and as it proved, in all cafes, as ufeful as could be wifhed, I preferred it to fulphuric acid, both of which are the principal arcana the difcovery of which I thought fo interefting to humanity. It is however to be wondered, how particularly the muriatic acid could have been fo long neglected, as the extent, and the abfolute neceffity of its ufe, in its combination with natron as common falt, fhould have long ago engaged the attention of medical men; and it is from the above experiments, and thefe theoretical arguments, that I am induced to allow, with refpect to medicinal quality, the preference to the muriatic acid; and though it has not yet been decompofed by a chemical procefs, it is, however, a matter of the higheft probability, that this is effected in the organic body, as neither the falt itfelf, nor the muriatic acid, is excerned from it in fubftance, after being internally taken.

"Befide the fulphuric and muriatic acids, which, with refpect to their intenfive ftrength, furpafs the reft, I have alfo made ufe of other acids for the fake of putting my principles to a fair trial, and it is with fatisfaction that I found them in this way confirmed. The nitric acid proved frequently very ufeful to me in dyfenteries and chronical painful diarrhœas; I do however but feldom employ it now, becaufe, befides its being much inferior to muriatic acid in point of volatility, it is imperfectly decompofed in the body, and only a part of oxygen difengaged from it, whereas the greateft portion remains in the body, combined with oxygen in form of nitrous acid, which merely differs in degree from that matter, which, which, according to the ingenious theory of Prof. Mitchill, ought to be efteemed the frequent fource of the moft dangerous epidemick difeafes. I obferved likewife, that confiderable fwellings often enfued the application of it. The phofphoric acid, which I have alfo fometimes ufed, did never effectually operate in urgent cafes; and as it is the moft fixed of all, little effect can in general be expected from it.

"The fuper-oxygenated muriatic acid, however, is one of the moft efficacious, agreeable, and cheapeft remedies, from the internal ufe of which, I have frequent inftances of the beft fuccefs, particularly when oxygen was required to be brought quickly to the organs, as in cafes of lethargy, &c. though it is by far inferior to fimple muriatic acid, which contains a greater intenfive proportion of oxygen than this. The vegetable acids have but little effect, becaufe they contain a great quantity of hydrogen and carbon in their mixture, but they may however prove of fome avail in flight febrile affections.

"The beft way of adminiftering all thefe acids, is to bring them immediately into the organs of digeftion; and the chief place of their action is the ftomach. They may, however, be applied with fuccefs in clyfters, and likewife externally on the fkin, in baths or fomentations, in both which cafes they ought alfo to be fufficiently diluted.

"But the following objection may be raifed againft the ufe of acids, according to the above theory, viz. that fevers are removed without any application of acids, by quite different remedies, and that confequently their ufe is not fo abfolutely neceffary as is here afferted, and that the whole conftruction of the above principles is therefore deftroyed. To this I anfwer in reply, that the known febrifuge remedies are either fubftances compound of mineral bodies and acids, or vegetable fubftances, that Nature has provided with more or lefs oxygen, in a free or bound ftate, which are decompofed into their elementary particles by the chemical and galvanic laws of the body. It may now be eafily conceived, how mineral bodies act againft fevers, and likewife vegetables that are endowed with free oxygen or acid; but how vegetable fubftances not containing any acid, are capable of repreffing a fever, is ftill a matter of inquiry and difcuffion. It ought however to be advanced here, that acid and oxygen are two things utterly different from each other, the firft being a compound, combuftible fubftance; the latter fimple, uncombuftible, and not to be reprefented by itfelf, whofe exiftence can only be conjectured, but not perceived by our fenfes. As the life of the human organic body continues only by the mutual action of oppofite principles, of which oxygen acts as the negative power, it is alfo probable, that this principle is according to the inconteftible laws of Nature, neceffarily requifite for vegetable organifm, and confequently contained in every vegetable fubftance in greater or lefs proportion. Thus, for inftance, the *bark* is, according to the refearches of Mr. Fourcroy, provided with a very confiderable portion of oxy-

 gen, gen, which alfo, after my experiments, is found in the different indigenous barks and woods that have been propofed as fubftitutes for it. Aromatic plants, refins, effential oils, fpirits, ethers, and the camphor, contain a great deal of oxygen; and opium even poffeffes much of it. The ftate and degree of oxydation in vegetables remain ftill to be afcertained by further refearches, but whether this may be performed through the medium of a retort, is very much to be queftioned. It is probable, that if thofe remedies do not operate in the body, by immediately imparting oxygen to it, they either combine themfelves there according to the laws of affinity with thofe fubftances, by which the oxygen was bound in it, which is in this way difengaged and left to operate, or they produce an alteration in the fyftem, by which the fame thing is effected. Thus may the action of the excitant remedies be explained, which are frequently of fuch great avail in the laft periods of fevers. But as it is in the power of Art to reftore the want of oxygen by remedies that immediately operate, it feems more proper rather to employ them, than to have recourfe to thofe that only have a mediate effect.

"It may in fome cafes however be advifable, for the fake of raifing and multiplying the affinity, to give alternately, or at the fame time, the mediately operating remedies with thofe that act immediately; but thefe cafes fhall be in future more fpecially determined. That emetics, purgatives, and clyfters, have fometimes a very good effect in fevers, by evacuating at once the matters, from which a new ftimulus febrilis is always evolving itfelf, can likewife not be denied. Baths alfo, and fomentations, contribute a great deal to the cure of fevers, by reducing or diminifhing the warmth according to their temperature with refpect to the body; but it has been above mentioned, how much their effect is heightened by the addition of acids.

"The quantity of acids internally requifite for the cure of a fever, cannot be well afcertained, and merely depends on the fuccefs; but intelligent phyficians will be able to adminifter them in fuch a manner, that the expected effect, the cure of fever, is obtained without injuring any organ neceffary for life.

"It has been laid down as a general rule in curing difeafes, always to adjuft the dofe of any remedy according to the different circumftances; that is to fay, according to the previous degree of fermentation. The above mineral acids ought therefore to be adminiftered, in beginning and increafing fevers, in a moderate dofe, often repeated, viz. from one drachm to half an ounce in eight ounces of water, and fome ounces of any fyrup; or if indicated, mixed with any fpirituous ftimulating fubftance; to be taken every hour or every two hours, from one to two table-fpoons full, or half a coffee cup, to which dofe water muft be mixed, or drank foon after. In time of danger, however, and in the period of crifis, one to two drachms, (40, 50, 60, to 100 drops) may be given at once, and repeated if thought proper. The fulphuric acid being ftronger than the muriatic and nitric acid, lefs is required quired of it than of thefe; the fuper-oxygenated muriatic acid however, may be adminiftered in large quantities, from one to two ounces every half hour or every hour. I have myfelf taken, in the courfe of four hours, a whole quart of it, and feveral of my patients took in the fame time twelve or more ounces, without experiencing any other fymptom than three or four watery ftools.

"Although the intenfive ftrength of any of thofe acids is not to be much attended to, as every thing depends on the event alone, and if 40 or 50 drops do not produce the expected effect, the dofe ought to be increafed or oftener repeated, yet it facilitates the whole proceeding, when acids of equal ftrength are always employed. How long the acids muft be continued is only determined by different circumftances; and while in fome cafes 100 drops are fufficient, others may perhaps require 800 drops. And the more the fever approaches to the higheft degree of fermentation, the lefs is wanted for its termination and cure

"Several inconveniencies accompany the ufe of mineral acids, which frequently oppofe unfurmountable difficulties to the phyfician. The very difagreeable tafte of acids can only be fomewhat remedied by fufficiently diluting and fweetening them with juices that are pleafant to the tafte. The forenefs of the lips and of the internal furface of the mouth, fometimes obferved, may generally be thought to be occafioned by not having taken fufficient care to involve and dilute them; but moft frequently it originates in the vehemence and malignity of the fever itfelf, that brings on a particular difpofition to fuch excoriations. There is nothing to fear for the ftomach on that account, when they are given with proper care; and they are indeed not more detrimental to the ftomach than pepper, horfe-radifh, muftard, &c. which fubftances, though caufing blifters on the external furface of the body, are taken without any harm internally. The difagreeable fenfation of the teeth, which is attended with the frequent ufe of acids, is of no great confequence, and difappears on the difcontinuance of them.

The fymptoms of a favourable event from the ufe of acids in fevers, differ according to the different kinds of thofe affections. Eructations, and even vomition, at the time of deglutition, gurgling of the bowels, difcharge of wind, diarrhœas, rifing and finking of the pulfe, change of heat and froft, fweats, falivation, increafed fecretion of urine, reft and fleep, &c. are generally the fymptoms from which, in urgent cafes, good fuccefs may be hoped. The moft favourable fymptom however is, when the fenfelefs patient awakes from his ftupor, and recovers his fenfes, or falls into a found fleep, in which the pulfe becomes flower, more regular, and moderately full.

"Deadly fymptoms generally were, according to my obfervations, when purple fpots appeared in the face, when one eye was half opened while the other was fhut in a paralytic manner; when the cornea became again opaque, after it had been previoufly rendered clear by the ufe of thofe remedies; when the patient falls back into fenfeleffnefs, attended with a facies hippocratica; when a rattling a rattling in the throat is perceived, and the pulfe becomes intermittent and irregular.

"After having removed all danger, and banifhed the fever by the acids, it is not abfolutely neceffary to employ other medicines, and I have, except in few cafes, given no other remedies, but left the entire recovery to Nature, affifted by a good and nourifhing diet."

Thefe are the chief principles of Dr. Reich's peculiar fyftem of fevers, which, as he pretends, will enable every practitioner to cure every kind of fever, under whatever name they may be recorded in the annals of medicine. He promifes to give a more copious and full explanation of them in a particular work, which will fhortly appear. However confcious of the imperfections that may be ftill imputed to his fyftem, he does not dread the trial of his principles, as he efteems them to be particularly calculated for uniting all parties and all fymptoms. Nobody, he thinks, whofe conceptions of a chemical procefs are not limited by grofs and vulgar ideas, will ever imagine, that by generally adopting the principles laid down here, the times of Sylvius are likely to return; but fully perfuaded of the neceffity of raifing the art of healing to that point of view, he fhould feel the utmoft fatisfaction, if the path were purfued he had attempted to tread.

Profeffor Reich proceeds now to point out the advantages which he pretends to refult from his difcovery for medicine, as well as for the community; and we fhall relate them, but without entering into their fpecial explanation, as he moftly appeals to his previous theoretical arguments. Thefe advantages are the following:—

"1. The medical fcience will thereby obtain a furer and firmer bafis, upon which a more accomplifhed fyftem may in future be conftructed, by farther obfervation than we have hitherto had.

"2. All febrile difeafes, without exception, will be contemplated from a better point of view, and, of courfe, more fpeedily and fuccefsfully removed; and every practitioner will be enabled, upon more rational grounds and principles, to difpel all danger during a few hours, in cafes where no particular complication exifts, nor the organs, neceffary for life, are too much hurt: Farther, by that method of cure, a fhorter duration of thofe difeafes will be occafioned, the moft generally bad and detrimental fymptoms prevented, and ftrength of the body in the eafieft and quickeft way reftored.

"3. Certain difeafes, which have hitherto been thought to be incurable, or very dangerous, and only in fome cafes cured by empiric remedies, will, according to thofe principles, be better underftood and more fuccefsfully treated." The author means by them the hydrophobia, the plague, yellow fever, and pulmonary confumptions; and though he has not had an opportunity of treating the three firft difeafes, he is convinced, by rational arguments and his fuccefs in other fevers, that acids, particularly when given in the firft periods of thofe difeafes, will have the moft fuccefsful effects. With refpect to confumptions, he afferts that he has faved the the life of many consumptive people, or, at least, considerably relieved them; and on that account, he recommends to his patients an acid diet, and orders them to drink fluids mixed with muriatic or sulphuric acid, and spiritus vini; in which way their strength and health were entirely restored, as far as the state of the lungs would admit of.

"4. Epidemic, putrid, or nervous fevers, diseases of camps and hospitals, by which thousands are destroyed, will in future be cured with security and less difficulty, and at the same time in the cheapest and most simple way.

"5. By the discovery of those remedies, and that new method of cure, field physicians will be enabled to prevent the rise and progress of such diseases, as far as this lies in the power of man." He recommends, therefore, to allow each soldier, on any long and wearisome march, a certain portion of elixir acidum Halleri.

"6. The mortality of the most destructive epidemic diseases of children, as measles, scarlet fevers, small-pox, hooping cough, &c. will be more securely and easily prevented than has hitherto been the case;" and, consequently,

"7. The great mortality of children will be greatly diminished by adopting those principles: The mortality of children in the infantile age, originates in the false ideas on the cause of the diseases to which they are exposed, as they are generally ascribed to an acrimony in the stomach, against which, alkaline and absorbent remedies were employed; but the little good they did, consisted in the carbonic acid that was, through their medium, brought into the body. The mineral acids, however, prove, according to Mr. Reich, of the greatest avail in all the different affections of the infantile age, difficult dentition, diarrhœas, convulsions, &c. The general prescription is, to mix with two ounces of any syrup and the same quantity of water, half a drachm to two drachms of spiritus vitrioli, or muriatic acid; half a drachm of liquor anodynus, or spiritus vini rectificatus, of which mixture, every hour, or every two hours, one or two table spoonfuls are to be taken; when the diseases are attended with much pain, some laudanum, or tincture of opium may be added.

"8. The treatment of several non-febrile diseases will, by that method, be considerably and successfully reformed.

"9. The remedies by which the cure is performed, are very cheap, and, at the same time, fevers are cured in a shorter period with less expence, whereby considerable sums will be saved, that are exported for foreign drugs."

Such is the Theory of Dr. Reich, and the method of cure which is grounded upon it. Although we may not deny that it agrees with the new discoveries in chemistry and natural philosophy, yet, it must stand the test of experience and observation before it is likely to meet with general approbation. Of twenty-eight patients who were treated by Dr. Reich in the Charité Hospital, under the superintendance of the Committee, three died, but the rest were cured. In private practice the remedies were used with different ferent succefs, and fome obftinate quartans and tertians were removed by them; while, in other cafes, they proved of little or no avail.

We fhall not omit communicating to our readers the further tranfactions relating to this theory, which are likely to be publifhed by different phyficians.

---

*An Essay on the Malignant Pestilential Fever, introduced into the West Indian Islands from Boullam, on the Coast of Guinea, as it appeared in* 1793, 1794, 1795, *and* 1796. *Interspersed with Observations and Facts, tending to prove that the Epidemic existing at Philadelphia, New York, &c. was the same Fever introduced by Infection imported from the West India Islands: And illustrated by Evidences founded on the State of those Islands, and the Information of the most eminent Practitioners residing on them.* By C. Chisholm, M. D. and Infpector General of the Ordnance Medical Department in the Weft Indies. Two vols. 8vo. about 1000 pages. London. Mawman. Price 16s. boards.

The Author, in his Preface to this fecond Edition, fays, "I have exhibited the motives which induced me to prefent the public with a ftatement of my opinion refpecting the nature, and of my reafons for adopting a new mode of treatment of the Malignant Peftilential Fever. Any further addrefs might feem unneceffary, and would, in truth, become fo, had not much, and in fome inftances illiberal, controverfy taken place, tending to fubvert that opinion, and to difcredit that treatment. How far the authority, on which refts the vehement oppofition of thefe controvertifts, may be relied on, is certainly an object of importance: But as no facts fupport them—as no local knowledge or information appear to have been acquired—and as their affertions feem to be founded on the bafelefs fabric of theory, and the generally received but falfe idea of the incompatibility of a heated and rarified atmofphere to retain infection—it muft neceffarily remain with me as vifionary, or, at beft, equivocal. And I am the more inclined to confider this oppofition in a light which reflects little credit or refpect on the authors of it, becaufe the refult of my own obfervation, and of the enquiries which I have induftrioufly made, during my later refidence in the Weft Indies, has only ferved to confirm me more ftrongly in the truth of what I have already offered on the fubject. But, as the public poffefs a right to more than fimple affertion on my part, I have judged it proper to intrude myfelf once more on their attention, and to offer my fentiments in a form more diffufed, and more capable of fixing the mind, than that of the firft Edition of my Effay on the Malignant Peftilential Fever.

"In the execution of this defign, I have thrown the following work into four parts. The firft contains an account of the origin, progrefs, diagnoftic, caufe, and other circumftances of the pathology of the difeafe; each of which is treated of in a diftinct chapter. The fecond relates the means of cure, in five chapters; the three firft are appropriated to the difcuffion of the three general indications dications of cure, the refult of the confideration of the circumftances peculiar to the difeafe. The fourth is employed in the difcuffion of the principles on which the efficacy of mercury in fevers of hot climates in general, and more efpecially in the Malignant Peftilential and Yellow Remittent Fevers, is founded. And the fifth is confined to fuch obfervations on the action of oxygenated medicines on the fyftem, deranged by fever and other morbid affections, in hot climates, as have occurred in a very extenfive ufe of them. For this, which, on a general view, feems to have little connexion with the fubject of the Effay, little apology perhaps is neceffary; and the late difcoveries relative to the fhare which the gafeous fluids have in almoft all curative intentions, may preclude the neceffity for any. The third part is altogether allotted to the important inveftigation of the means of prevention. In the courfe of this enquiry a queftion of infinite confequence is ftarted, and an attempt made to difplay the means by which the purpofe of it may be fulfilled; I mean how far the European conftitution may be prepared for the procefs of affimilation to the tropical climates?

"Having thus confidered the great outlines of thofe divifions of the following work which relate to the pathology, the cure, and the prevention of the Malignant Peftilential Fever, I fhall proceed to account for the publication of the fourth part.

"The oppofition which I have had, and have to contend with, in my endeavour to imprefs the truth of my fentiments on the public mind, relative to the origin and caufe of the propagation of the peftilential infection, which has characterifed the late direful epidemic; and to the mode of treatment which I, as well as every unprejudiced practitioner in the Weft India Iflands, have found the only fuccefsful one, has proceeded from the agents of the Bulama Affociation in the firft inftance, and from the Medical Staff of the Armies acting in the Weft Indies fubfequent to the year 1795. The attempts at refutation of the ftrong facts which have occurred at Grenada, by the agents of the Bulama Affociation, are fimilar to the affertions made by the writers for the Levant Company, againft the importation of infection into Great Britain immediately from Turkey. The firft have imagined that their colonies on the coaft of Africa might be injured; and the latter had fears of their trade fuffering: we fee therefore in both, intereft overleaping every obftacle, and fhutting the door againft truth. The conduct of the gentlemen compofing the medical ftaff cannot be fo obvioufly accounted for Was it neceffary to exhibit caufes for the wide devaftation which took place in the military hofpitals, we might expect a delineation of facts tending to prove that this proceeded from the faithful obfervation of the method recommended by me; but what fhall we fay, when we are told that the efficacy, and even danger, of this method, were inculcated at the commencement of the operations of the armies, and before cafes occurred on which trials might be made. It is not my intention to enter on the invidious task of difplaying the real caufes of the lofs of 13,437 foldiers, our countrymen, in a period little exceeding thirty months, by the Ma-

 lignant lignant Peſtilential and Yellow Remittent Fever; but I may, without incurring cenſure, ask, why the mercurial treatment was not recurred to, when that adopted had ſo generally failed? No ſatisfaction will ariſe from being told, that much of the dreadful mortality which took place proceeded from the latter of theſe diſeaſes; for the applicability of the queſtion to the nature of the event is not diminiſhed. I ſhould not, however, conſider it as neceſſary to ſtate the general circumſtances of the medical conduct of the Hoſpitals, did not the accumulations of death from 1796 to 1798, preſent an argument to the uninformed, againſt the utility and propriety of the mercurial treatment of the fatal maladies in queſtion. To ſuch the evidences which the fourth part compriſes may be uſeful; for my own juſtification I conſider them as neceſſary. A vain and abſurd attempt was indeed made in the year 1797, to ſhew that the exhibition of mercury, in the fatal epidemics which prevailed during that and the preceding year, had been generally recurred to; and that the general reſult was far from favourable; but the reports on which this belief was founded, were of ſuch a nature as diſcredited the poſition which they were intended to eſtabliſh; for it is univerſally known, that the mercurial treatment was induſtriouſly condemned by thoſe placed in the direction of the hoſpitals; and a youthful, inexperienced, and docile ſtaff readily truſted, where attention, application, and a denial of accuſtomed indulgence, would have been the price of ſucceſs. Were there a poſſibility of inaccuracy in this ſtatement, I would rejoice in ſuppreſſing it. I am perfectly open to conviction, and feel no enmity to any individual of the ſtaff; but juſtice to myſelf, and humanity, ſhuddering at the deſtruction of nearly the whole of the Weſt India army, call upon me to declare that there is undoubted authority for ſtating, that notwithſtanding the total inefficacy of the remedies generally employed in the general hoſpitals, the practice deſcribed by me was not recurred to by more than three army phyſicians in a fair and judicious manner. Two of theſe gentlemen are unhappily no more; but Dr. Wright is a living monument of the advantages which reſult from the employment of mercury, conducted by profeſſional ſkill, and by judgment. This gentleman's report has been made public, and the world unites in the eulogium of the author.

"The abſurd conduct of the Medical Board at home, at the time Sir Ralph Abercromby's army was formed, is chiefly reprehenſible; and may indeed, very juſtly, be conſidered as an extenuation of the inexperience, the folly, or the crime, of the younger members of the medical ſtaff of that army. In the name of common ſenſe, let me aſk, what uſeful purpoſes could ariſe from a regulation which precluded all from exerciſing the important functions of an army phyſician, but ſuch as derived their medical knowledge from ſeminaries where none can be obtained; or ſuch as were, or became licentiates of the London College of Phyſicians? What had an Oxford or a Cambridge degree; what had a licence to practiſe within ſeven miles of London, to do with medical cal practice within the tropics? Can the whole of that learned body united, communicate that experience in the treatment of inter-tropical fevers, which can be acquired only by a long and painful attention to them in the torrid regions where they are endemic? This singular infatuation, by which the order of things was inverted, proceeded from views best known to the triumvirate themselves; but its effects may be considered as the remote cause of all the misfortunes which befel the devoted troops composing the army under the command of Sir Ralph Abercromby. Our country, therefore, have inexpugnable reasons for consigning a Board, whose resolves and regulations militate so forcibly against the sober dictates of common sense, and whose self-sufficiency has given existence to a cloud which obscures the most manifest proofs of the baleful tendency of their measures, to the fate of the unhappy victims who have fallen under the operation of both.

"The fourth part of the following work is intended to constitute, therefore, a body of evidence, to shew the fallacy of the statements and reasoning of the Agents of the Bulama Association; and to exhibit the disingenuous conduct of the medical staff, and the fatal consequences of a disbelief of the efficacy of a remedy which had been established by the experience of a multitude of eminent practitioners in all the West India Islands, where the calamity it was calculated to correct, existed. In treating this part of my subject, the views of the actual state of each colony, I have given, may be considered as too diffuse, and unnecessarily minute; but I have found the matter which presented itself, important; and the shades of variation are too considerable to remain unexamined in the investigation of morbid causes. Those objects which are common to all, have been sufficiently explored in the Introduction to the first Edition, which I have therefore given a place in this.

"Dr. Trotter, of the Navy, has thrown out Remarks, the tendency of which is, to invalidate the authority on which I found my opinion relative to the origin, the nature, and the treatment of the Malignant Pestilential Fever. It would be with no uncommon concern I should enter the lists of controversy with a gentleman whose liberality of thinking, professional skill, and exertions in the cause of suffering humanity, have placed high in the estimation of the public; and I trust that a more attentive consideration of the facts and reasoning I have submitted to the public in the following work, will remove that inclination to differ from me, which is only too apparent in several passages of his excellent work, The Medicina Nautica."

